# Curcumin Analog, HO-3867, Induces Both Apoptosis and Ferroptosis via Multiple Mechanisms in NSCLC Cells with Wild-Type p53

**DOI:** 10.1155/2023/8378581

**Published:** 2023-02-13

**Authors:** Ling Wu, Guodong Xu, Ni Li, Linwen Zhu, Guofeng Shao

**Affiliations:** ^1^School of Medicine, Ningbo University, Ningbo, Zhejiang 315211, China; ^2^Department of Cardiothoracic Surgery, Lihuili Hospital Affiliated to Ningbo University, Ningbo, Zhejiang 315000, China

## Abstract

Over the last decade, researchers have paid more and more attention to the natural compound curcumin for its potential application in anticancer therapy. However, the application of curcumin has been limited owing to its rapid metabolism in the body. HO-3867, a stable curcumin analog, shows potent antitumor activities against various tumor cells. Yet, information on HO-3867's impact on non-small-cell lung cancer (NSCLC) cells is lacking. Herein, we evaluated the cytotoxicity of HO-3867 in NSCLC cells. We discovered that HO-3867 suppressed the viability of NSCLC cells containing wild-type p53. In NSCLC cells, HO-3867 promotes both apoptosis and ferroptosis, the latter of which is a newly discovered mode of cell death. Mechanically, HO-3867-induced apoptosis relied on the inhibition of Mcl-1 and Bcl-2 and the upregulation of Bax. Moreover, NSCLC cells undergo ferroptosis when treated with HO-3867 via activating the p53-DMT1 axis and suppressing GPX4. Additionally, HO-3867 caused an accumulation of reactive oxygen species (ROS) in NSCLC in a way that was dependent on the presence of iron. Our findings point to the possibility that HO-3867 might be employed as a therapeutic agent for treating NSCLC.

## 1. Introduction

Around 2 million new cases of lung cancer are diagnosed each year, with 1.8 million deaths globally due to the disease [1]. Histopathologically, non-small-cell lung cancer (NSCLC) accounts for the vast majority of lung cancer diagnoses (representing 80–85% of all cases) [2]. Up to this point, the primary therapeutic choices for NSCLC have been surgery, radiation, chemotherapy, and immunotherapy [3]. The overall prognosis of NSCLC individuals is still unfavorable, despite the evolving advances that have been achieved in the treatment of NSCLC. As a consequence, innovative effective therapeutic techniques are urgently required.

Programmed cell death (PCD) alludes to the death of cells in an organized manner that is meticulously modulated by a wide range of endogenous genes. PCD is implicated in a variety of physiological activities such as tumorigenesis. The most studied form of PCD is apoptosis mainly hallmarked by chromatin condensation, cellular shrinkage, DNA fragmentation, formation of apoptotic body, and membrane blebbing [4]. The apoptotic process is controlled by numerous proteins, such as those belonging to the Bcl-2 family [5]. Unlike apoptosis, necroptosis, and autophagy, the recently discovered ferroptosis is a distinctive PCD mechanism [6]. Ferroptosis is hallmarked by the formation of lipid peroxides in a process dependent on the presence of iron, which subsequently promotes the accumulation of reactive oxygen species (ROS) in cells and, ultimately, results in the death of cells as a result of oxidative stress [7]. Mounting data indicate that defective ferroptosis is implicated in carcinogenesis [8]. Therefore, triggering ferroptosis is a viable technique for eliminating cancer cells, particularly those that are insensitive to apoptosis [9].

In past decades, the strong potential of natural products to destroy cancer cells while exhibiting relatively low toxicity has garnered considerable interest in cancer therapy. Curcumin, the active ingredient of *Curcuma longa* L, has been found to exhibit anticancer actions against multiple cancers, one of them being lung cancer [10]. Although curcumin is safe even at large dosages in humans, its rapid metabolism in the body has limited its clinical use [11]. To improve the outcomes, various adjuvants and structural analogs of curcumin have been developed. Among them, the new diarylidenyl piperidone (DAP)-inspired analog HO-3867 promotes growth arrest and apoptosis in a myriad of different tumor cell lines, even those from breast, colon, liver, lung, and ovarian cancers, without evident toxicity to normal cells [12, 13].

Nevertheless, research on HO-3867's impact on NSCLC cells has not been extensively conducted so far. Therefore, in this research, we systematically examined the mechanisms behind the antitumor effects of HO-3867 on NSCLC cells.

## 2. Materials and Methods

### 2.1. Chemicals

HO-3867 (cat no: S7501), DFO (cat no: S6849), Dp44mT (cat no: S790), dexrazoxane HCl (cat no: S5651), salinomycin (cat no: S8129), ebselen (cat no: S6676), z.VAD.FMK (cat no: S7023), Nec-1 (cat no: S8037), Fer-1 (cat no: S7243), Lip-1 (cat no: S7699), and PFT*α* (cat no: S2929) were procured from Selleck Chemicals (USA). S-Benzylisothiourea (cat no: S9138) was ordered from Sigma-Aldrich (USA). The primary antibodies listed below were ordered from Cellular Signaling Technology (USA): caspase-3 (cat no:9662), Mcl-1 (cat no:94296), Bcl-2 (cat no:15071), Bcl-xL (cat no:2762), Bak (cat no:6947), Bax (cat no:94296), and DMT1 (cat no:15083). The following primary antibodies listed were procured from Abcam (USA): FPN1 (cat no: ab239583), TFR (cat no: ab214039), FTL (cat no: ab75973), FTH (cat no: ab183781), and p53 (cat no: ab26). The secondary antibody was procured from Abclonal (China). Sigma-Aldrich supplied all other routine chemicals.

### 2.2. Cell Culture

The American Type of Culture Collection (ATCC, USA) supplied the human NSCLC cell lines (H460, PC-9, H1975, A549, and H1299). A549 p53 KO cells (cat no: ab276092) was ordered from Abcam. H460 p53 KO cells were a generous gift from Dr. Qingfeng Li (Fudan University, China). The STR test was used to verify the authenticity of the cell lines, and mycoplasma testing was performed routinely. The culture medium comprised RPMI1640 with 10% fetal bovine serum (FBS, Gibco, USA) and 100 U/ml penicillin/streptomycin (Sigma-Aldrich). The cells were maintained in an environment containing 5% CO_2_ at a temperature of 37°C.

### 2.3. Cell Transfection

GenePharma Ltd. (Suzhou, China) supplied the siRNAs for Bax and DMT1 knockdown. The following sequences were applied: DMT1 #1 siRNA: 5′-CCTAAAGTGGTCACGCTTTTT-3′, DMT1 #2 siRNA: 5′-ACCTCGGAGGAATCGTTAGA-3′, Bax #1 siRNA: 5′-AGCTGGATGGCAATGTTGAGCT-3′, and Bax #2 siRNA: 5′ -AAGTTCCTGATATTATCAG-3′. Overexpression of Bcl-2, Mcl-1, and GPX4 was achieved by subcloning their respective full-length cDNAs into pcDNA3.1 (GenePharma Ltd). An empty vector was used as a negative control. In addition, Lipofectamine 2000 (Life Technologies, USA) was used for the transfection following package instructions. Western blots were employed to assess the effectiveness of the transfection.

### 2.4. Cell Viability Assay

The MTT test was employed to ascertain the cell viability, as earlier reported [14]. For 12 h at 37°C, the cells were inoculated onto 96-well plates at a density of 3 × 10^4^ cells/mL, with five parallel wells in each group. After that, the cells were subjected to HO-3867 treatment in a variety of concentrations and timeframes (24 h, 48 h, and 72 h). Next, following the addition of 10 *μ*l of MTT to each well, they were allowed to incubate at 37°C for additional two hours. The findings were presented in the form of a ratio to the absorbance value recorded at 570 nm for the cells serving as controls. There were three separate repetitions for the experiment.

### 2.5. Cell Death Measurement

The Annexin V-FITC Apoptosis detection kit (BD Bioscience, USA) was adopted to measure cell death following the directions stipulated by the manufacturer. Succinctly, cells were harvested at various time points following treatment followed by the resuspension of precipitate in 400 *μ*l 1 × binding buffer. After that, 5 *μ*l Annexin V-FITC/PI was introduced into the cells, and then, they were allowed to incubate for 20 min at room temperature (RT) in the darkness. For each assessment, 1 × 10^4^ cells were counted and examined via flow cytometry (BD Biosciences, USA) to determine cell death.

### 2.6. Caspase-3 Activities Assay

Caspase-3 Activity Assay Kit (cat no: ab252897, Abcam) was employed to determine the relative activities of caspase-3 activity, as instructed by the manufacturer.

### 2.7. Quantitative RT-PCR

Through the use of the TRIzol reagent (Beijing Solarbio Science and Technology Co., Ltd.) as directed by the manufacturer, total RNA was isolated from the cells. The levels of messenger RNA (mRNA) produced by distinct genes were measured with the assistance of a first-strand cDNA synthesis kit (cat no: K1611) and SYBR Green Master Mix kit (cat no: A25741, Applied Biosystems; Thermo Fisher Scientific, Inc.) in conformity with the instructions stipulated by the manufacturer. The ABI 7500 Fast real-time PCR system (Applied Biosystems; Thermo Fisher Scientific, Inc.) was employed for the qRT-PCR procedure. For thermocycling, we applied the following procedures and parameters: initially, the protein was denatured at 95°C for 10 minutes, and then, it was subjected to 35 cycles for 15 seconds at 95°C, 30 seconds at 55°C, 30 seconds at 72°C, and a final extension for 5 mins at 72°C. Glyceraldehyde 3-phosphate dehydrogenase (GAPDH) expression was measured and it served as an internal control.

### 2.8. Iron Level Assessment

An iron assay kit (ab83366, Abcam) was utilized to determine relative Fe^2+^ levels in compliance with the specifications provided by the manufacturer. Succinctly, overnight cultures were started from cells (1 × 10^5^ cells/well) sown in 6-well plates. After that, different treatments were applied to the cells for 24 hours. Cells were homogenized with 100 *μ*L iron assay buffer and centrifuged at a rate of 13,000 × g for 10 min at 4°C, and the pellet was then discarded. To prevent the transformation of Fe^3+^ to Fe^2+^, the iron reducer included in the kit was introduced into the supernatant. The components were stirred carefully and left to incubate for 30 minutes in the darkness on ice. After adding 100 *μ*L of Fe^2+^ probe, samples were allowed to incubate for 1 hour at RT, away from light. A microplate reader (BioTek, USA) was employed to measure the absorbance at 593 nm. There were three separate repetitions for the experiment.

### 2.9. Assessment of ROS

A ROS detection assay kit (ab287839, Abcam) was employed to determine ROS levels in compliance with the instructions from the manufacturer. In brief, cells (1 × 10^5^ cells/well) were cultured throughout the night using 6-well plates. After that, the cells were then subjected to various treatments for 24 h. For 30 minutes at 37°C, cells were exposed to 10 *μ*M DCFH-DA in FBS-free media before being rinsed thrice. The ROS level was determined by flow cytometry (BD Biosciences, USA) with cell counts of 1 × 10^4^ cells for each measurement.

### 2.10. Measurement of Glutathione (GSH)

A GSH assay kit (cat no: ab239727, Abcam) was employed to determine the relative level of GSH as per the package recommendation. In brief, 1 × 10^5^ cells/well were seeded into 6-well plates and allowed to incubate in the darkness for 12 hours. Different treatments were applied to the cells for 24 hours. After homogenizing the cells in a solution containing 50 mM MES and 1 mM EDTA, the cells were centrifuged at a rate of 13,000 x g for 10 minutes at 4°C followed by the removal of the pellet. After mixing the supernatant with the GSH detection solution and incubating for 25 minutes at RT, the results were read. After transferring the reaction solution to a 96-well plate, the microplate reader (BioTek, USA) was used to read the findings at a wavelength of 412 nm. There were three separate repetitions for the experiment.

### 2.11. Western Blots

The RIPA lysis buffer (Beyotime, China) was utilized in the preparation of total cell lysates. The BCA Protein Assay Kit (Beyotime, China) was applied to determine the concentrations of protein as directed by the manufacturer. The proteins were extracted using an SDS-PAGE gel at a concentration of 10%, and then, they were transferred onto a PVDF membrane (BD Biosciences). After blocking the membrane using nonfat milk at a concentration of 5%, it was allowed to incubate with primary antibodies for a full night at 4°C. After being rinsed in PBS thrice, the membrane was allowed to incubate in an incubation solution containing the necessary secondary antibody for one hour at RT. The enhanced chemiluminescence (ECL) system (Merck Millipore, Darmstadt, Germany) was utilized to facilitate the visualization of the findings.

### 2.12. Statistical Analysis

The software SPSS 25.0 (IBM, USA) was employed to execute all analyses of statistical data. The data were reported using mean ± SD. We made a comparison between the two groups with the unpaired two-tailed Student's *t*-test, and comparisons among multiple subgroups were carried out with a one-way analysis of variance (ANOVA), followed by Tukey's post-hoc test. A *P* value of <0.05 (two-tailed) indicated the significance level.

## 3. Results

### 3.1. HO-3867 Suppressed the Viability of NSCLC Cells with Wild-Type p53

**Figure 1:**
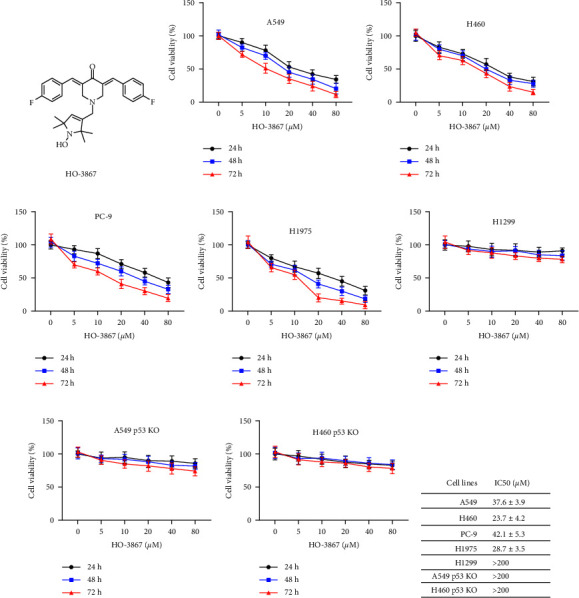
HO-3867 inhibited the viabilities of NSCLC cells with p53 wild-type. (a) The chemical structure of HO-3867. (b) A549 cells were treated with various doses of HO-3867 for the indicated time (24 h, 48 h, and 72 h), and cellular viabilities were measured. (c) H460 cells were treated with various doses of HO-3867 for the indicated time (24 h, 48 h, and 72 h), and cellular viabilities were measured. (d) PC-9 cells were treated with various doses of HO-3867 for the indicated time (24 h, 48 h, and 72 h), and cellular viabilities were measured. (e) H1975 cells were treated with various doses of HO-3867 for the indicated time (24 h, 48 h, and 72 h), and cellular viabilities were measured. (f) H1299 (p53 deficiency) cells were incubated with various doses of HO-3867 for the indicated time (24 h, 48 h, and 72 h), and cellular viabilities were measured. (g) A549 p53 KO cells were incubated with various doses of HO-3867 for the indicated time (24 h, 48 h, and 72 h), and cellular viabilities were measured. (h) H460 p53 KO cells were treated with various doses of HO-3867 for the indicated time (24 h, 48 h, and 72 h), and cellular viabilities were measured. (i) IC50 of HO-3867 in different NSCLC cells. Data are presented as mean ± SD.

Figure 1(a) depicts the chemical structure of HO-3867. Various NSCLC cell lines (H460, PC-9, H1975, A549, and H1299) were subjected to increasing dosages of HO-3867 (5, 10, 20, 40, and 80 *μ*M) at distinct timeframes (24 h, 48 h, and 72 h), and the MTT test was employed to assess the viability of the cells. HO-3867 suppressed the viability of NSCLC cells with p53 wild-type (A549, H460, PC-9, and H1975) in a time- and dosage-dependent way (Figures 1(b)–1(e)). The HO-3867 treatment had almost no impact on the p53-deficient NSCLC cell line's (H1299) viability (Figure 1(f)). For subsequent evaluation of the influence of p53 in the cytotoxicity of HO-3867 against NSCLC cells, two p53 KO cell lines (A549 p53 KO and H460 p53 KO) were treated with HO-3867. As indicated in Figures 1(g) and 1(h), neither A549 p53 KO nor H460 p53 KO cells were sensitive to HO-3867. Calculation of IC50 values confirmed that H1299, A549 p53 KO, and H460 p53 KO cells are less sensitive to HO-3867 than NSCLC cell lines with wild-type p53 (Figure 1(i)). Collectively, these findings illustrated that the cytotoxic action of HO-3867 against NSCLC cells relies on p53.

### 3.2. NSCLC Cells Undergo Caspase-Dependent Apoptosis When Treated with HO-3867

**Figure 2:**
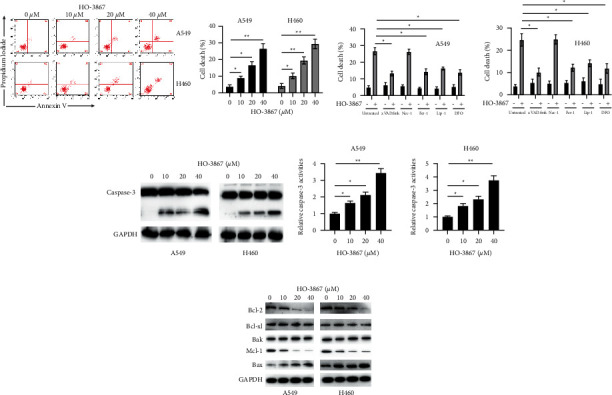
HO-3867 induces apoptosis in NSCLC cells. (a) A549 and H460 cells were incubated with the indicated doses of HO-3867 for 24 h, and cellular death was measured by flow cytometry. (b) A549 and H460 cells were incubated with 40 *μ*M of HO-3867 in combination with different cell death inhibitors (z.VAD.FMK: 5 mM, Nec-1: 5 mM, Fer-1: 5 mM, Lip-1: 5 mM, and DFO: 10 mM) for 24 h, and cellular death was measured. (c) A549 and H460 cells were incubated with the indicated doses of HO-3867 for 24 h, and caspase-3 levels were measured by Western blotting. (d) A549 and H460 cells were incubated with indicated doses of HO-3867 for 24 h, and caspase-3 activities were measured. (e) A549 and H460 cells were incubated with various doses of HO-3867 for 24 h, and the indicated proteins were measured by Western blotting. Data are presented as mean ± SD. ^*∗*^*P* < 0.05 and ^*∗∗*^*P* < 0.01.

Cell death in NSCLC was studied to assess whether it may be triggered by HO-3867. Varying concentrations of HO-3867 (10 *μ*M, 20 *μ*M, and 40 *μ*M) were employed to treat H460 and A549 cells for 24 h. In addition, Annexin V-FITC staining was employed to measure cell death. Cell death in NSCLC was dosage-dependently promoted by HO-3867 (Figure 2(a)). Several cell death inhibitors (z.VAD.FMK, Nec-1, Fer-1, and Lip-1) were employed to confirm the specific mode of cell death produced by HO-3867. As depicted in Figure 2(b), both ferroptosis inhibitors (Fer-1, Lip-1) and apoptosis (z.VAD.FMK) remarkably suppressed the HO-3867-induced cell death. Moreover, the iron chelator deferoxamine (DFO) also suppressed HO-3867-induced cell death, and cell death caused by HO-3867 was unaffected by the necroptosis inhibitor necrostatin-1 (Nec-1) (Figure 2(b)). To additionally establish that HO-3867 triggered apoptosis in NSCLC cells, caspase-3 activation was tested by Western blotting and caspase-3 activity assay. As indicated in Figures 2(c) and 2(d), caspase-3 activation in NSCLC cells was dosage-dependently stimulated by HO-3867. Since Bcl-2 family proteins perform an instrumental function in regulating apoptosis, we evaluated their levels after being treated with HO-3867. Treatment with HO-3867 decreased Mcl-1 and Bcl-2 and increased Bax in NSCLC cells (Figure 2(e)). Collectively, these findings suggest that HO-3867 induces both apoptosis and ferroptosis in NSCLC cells.

### 3.3. Overexpression of Mcl-1 and Bcl-2 or Downregulation of Bax Reduced HO-3867-Induced Apoptosis in NSCLC Cells

**Figure 3:**
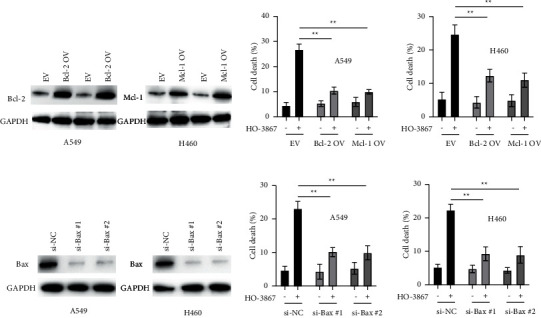
Overexpression of Bcl-2/Mcl-1 or knockdown of Bax suppressed HO-3867-induced cell death in NSCLC cells. (a) A549 and H460 cells were transfected with empty vector (EV), or Bcl-2 overexpressing vector (Bcl-2 OV), or Mcl-1 overexpressing vector (Mcl-1 OV) for 24 h, and total proteins were subjected to Western blotting using the indicated antibodies. (b) A549 and H460 cells were transfected as indicated for 24 h cells were treated with or without HO-3867 for another 24 h and cellular death was measured. (c) A549 and H460 cells were transfected with negative controls siRNA (si-NC) or siRNAs against Bax (si-Bax#1 and si-Bax#2) for 24 h, and protein levels of Bax were measured. (d) A549 and H460 cells were transfected as indicated for 24 h, cells were treated with or without HO-3867 for another 24 h, and cellular death was measured. The data were presented as mean ± SD. ^*∗∗*^*P* < 0.01.

After observing that HO-3867 influenced the expression of Bax, Mcl-1, and Bcl-2 in NSCLC cells, we overexpressed Mcl-1 and Bcl-2 in NSCLC cells to determine their involvement in HO-3867-induced apoptosis (Figure 3(a)). For NSCLC cells, overexpression of Mcl-1 or Bcl-2 significantly attenuated HO-3867-induced apoptosis (Figure 3(b)). The Bax gene was silenced in NSCLC cells (Figure 3(c)). Bax knockdown remarkably mitigated HO-3867-induced apoptosis in NSCLC cells, as depicted in Figure 3(d). Therefore, the decrease in Mcl-1 and Bcl-2 expression and the upregulation of Bax are crucial for the death of NSCLC cells triggered by HO-3867.

### 3.4. HO-3867 Induces Accumulation of Iron in NSCLC Cells

**Figure 4:**
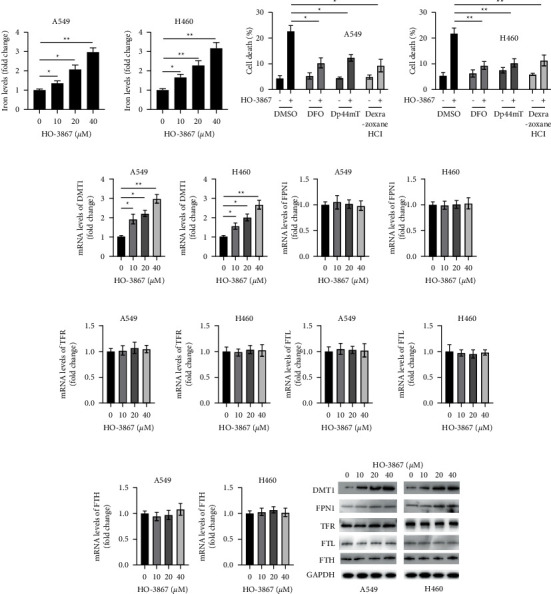
HO-3867 induced iron accumulation in NSCLC cells. (a) A549 and H460 cells were incubated with the indicated doses of HO-3867 for 24 h, and iron levels were assayed. (b) A549 and H460 cells were incubated with 40 *μ*M of HO-3867 in combination with different iron chelators (DFO: 20 *μ*M, Dp44mT: 20 *μ*M, and dexrazoxane HCl: 15 *μ*M) for 24 h, and cellular iron levels were assayed. (c) A549 and H460 cells were incubated with the indicated doses of HO-3867 for 24 h, and the mRNA levels of DMT1 were assayed by RT-PCR. (d) mRNA levels of FPN1. (e) mRNA levels of TFR. (f) mRNA levels of FTL. (g) mRNA levels of FTH. (h) A549 and H460 cells were incubated with the indicated doses of HO-3867 for 24 h, and total cellular lysates were subjected to Western blotting using the indicated antibodies. Data are presented as mean ± SD. ^*∗*^*P* < 0.01 and ^*∗∗*^*P* < 0.01.

Ferroptosis inhibitors were shown to reduce HO-3867-induced cell death; therefore, we assessed the involvement of HO-3867 in ferroptosis induction in NSCLC cells. We found that HO-3867 dosage-dependently induced iron accumulation in NSCLC cells (Figure 4(a)). Multiple iron chelators (DFO, Dp44mT, and dexrazoxane HCl) were used to verify iron's involvement in HO-3867-triggered cell death. As depicted in Figure 4(b), iron chelators remarkably reduced HO-3867-triggered ferroptosis in NSCLC cells. The expression of genes involved in iron transport in NSCLC cells was assessed using RT-PCR to elucidate the mechanism of HO-3867-induced ferroptosis. RT-PCR revealed that HO-3867 treatment induced the upregulation of mRNA levels of *DMT1* but not *FPN1*, *TFR*, *FTL*, and *FTH* in NSCLC cells (Figures 4(c)–4(g)). Western blotting confirmed the dosage-dependent upregulation of DMT1 by HO-3867 in NSCLC cells (Figure 4(h)). Therefore, HO-3867 induced iron-dependent cell death by upregulating DMT1 levels in NSCLC cells.

### 3.5. Upregulation of DMT1 in a p53-Dependent Way Is Critical for HO-3867-Mediated Ferroptosis in NSCLC Cells

**Figure 5:**
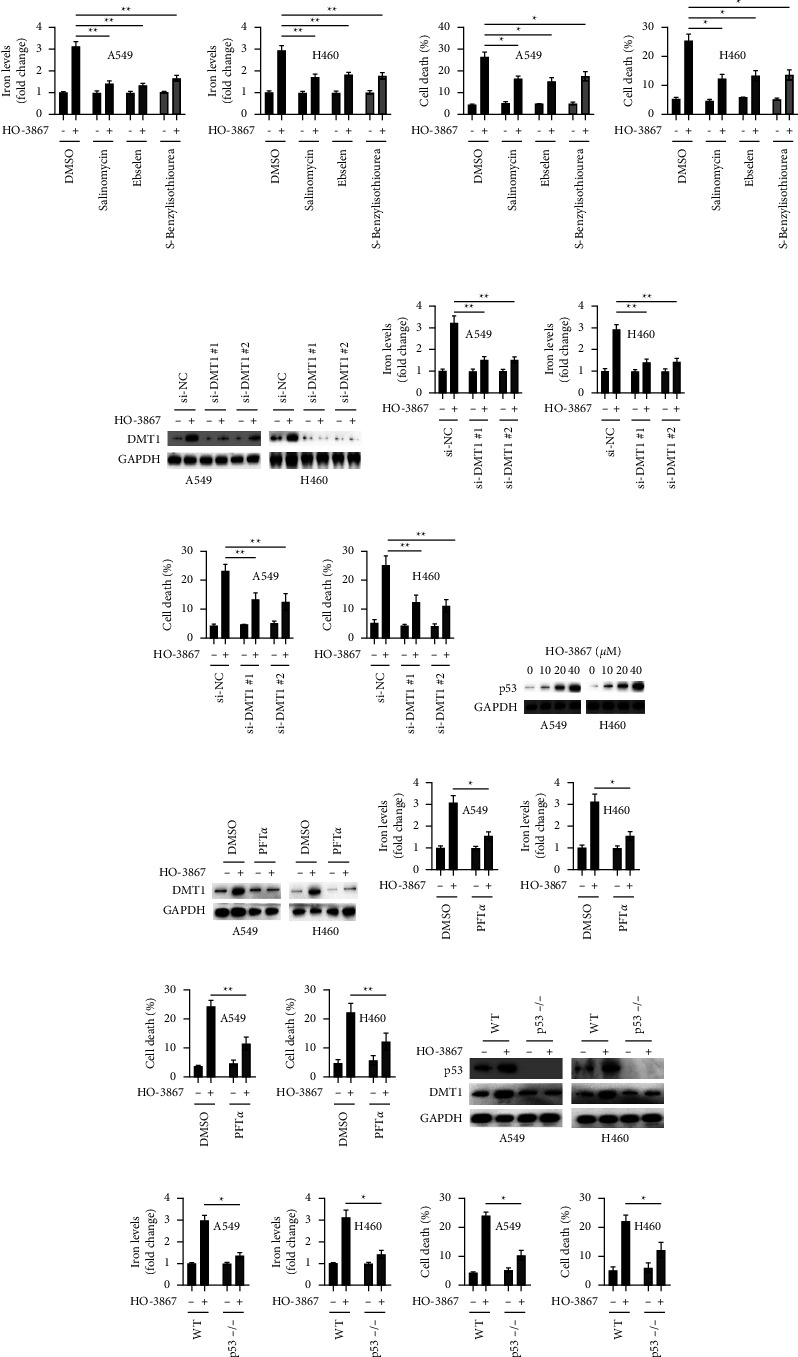
HO-3867-induced upregulation of DMT1 relied on p53 in NSCLC cells. (a) A549 and H460 cells were incubated with 40 *μ*M of HO-3867 in combination with different DMT1 inhibitors (salinomycin: 10 *μ*M, enselen: 20 *μ*M, and S-benzylisothiourea: 20 *μ*M) for 24 h, and levels of iron were assayed. (b) A549 and H460 cells were treated as above, and cellular death was assayed. (c) A549 and H460 cells were transfected as indicated for 24 h, cells were incubated with or without HO-3867 (40 *μ*M) for another 24 h, and protein levels of DMT1 were assayed by Western blotting. (d) A549 and H460 cells were treated as above, and iron levels were assayed. (e) Cellular death was measured. (f) A549 and H460 cells were incubated with indicated doses of HO-3867 for 24 h, and protein levels of p53 were assayed by Western blotting. (g) A549 and H460 cells were incubated with HO-3867 (40 *μ*M) in combination with PFT*α* (10 *μ*M) or not for 24 h, and protein levels of DMT1 were assayed by Western blotting. (h) A549 and H460 cells were treated as above, and iron levels were assayed. (i) Cellular death was assayed. (j) A549 and H460 wild-type (WT) or p53 KO (p53-/-) cells were treated with or without HO-3867 (40 *μ*M) for 24 h, and the indicated proteins were assayed by Western blotting. (k) Cells were treated as above, and iron levels were assayed. (l) Cellular death was assayed. Data are presented as mean ± SD. ^*∗*^*P* < 0.01 and ^*∗∗*^*P* < 0.01.

Several different DMT1 inhibitors (salinomycin, ebselen, and S-benzylisothiourea) were used to determine whether or not DMT1 was implicated in the HO-3867-triggered cell death. As illustrated in Figure 5(a), DMT1 inhibitors successfully abrogated the upregulation of HO-3867-induced iron in NSCLC cells. DMT1 inhibitors also remarkably suppressed HO-3867-mediated cell apoptosis in NSCLC cells (Figure 5(b)). Two siRNAs against DMT1 were used to silence DMT1 in NSCLC cells, allowing for additional investigation into DMT1's function in HO-3867-triggered cell death (Figure 5(c)). The downregulation of DMT1 also reduced the HO-3867-induced increase in iron levels in NSCLC cells (Figure 5(d)). *DMT1* silencing also remarkably suppressed HO-3867-mediated apoptosis in NSCLC cells (Figure 5(e)). As we noticed that p53-deficient cells are insensitive to HO-3867, we assessed the status of p53 in NSCLC cells. Results illustrated that HO-3867 administration resulted in a dosage-dependent upregulation of p53 in NSCLC cells (Figure 5(f)). The p53 inhibitor PFT*α* was employed to examine the link between p53 and DMT1. PFT*α* abrogated the upregulation of DMT1 induced by HO-3867 in NSCLC cells (Figure 5(g)). PFT*α* also reduced iron levels in NSCLC cells after exposure to HO-3867 (Figure 5(h)). Moreover, PFT*α* remarkably suppressed HO-3867-mediated death in NSCLC cells (Figure 5(i)). Thereafter, we assessed the status of DMT1 in p53 KO cells after exposure to HO-3867.  Figure 5(j) demonstrates that treatment with HO-3867 attenuated the upregulation of DMT1 in p53 KO cells. In addition, the upregulation of iron levels was also attenuated in p53 KO cells (Figure 5(k)). HO-5637-mediated cell death was also much less in p53 KO cells than in NSCLC cells with wild-type p53 (Figure 5(l)). Taken together, these findings indicate that HO-3867 upregulates DMT1 in NSCLC cells in a p53-dependent way.

### 3.6. HO-3867 Iron-Dependently Induced Accumulation of ROS in NSCLC Cells

**Figure 6:**
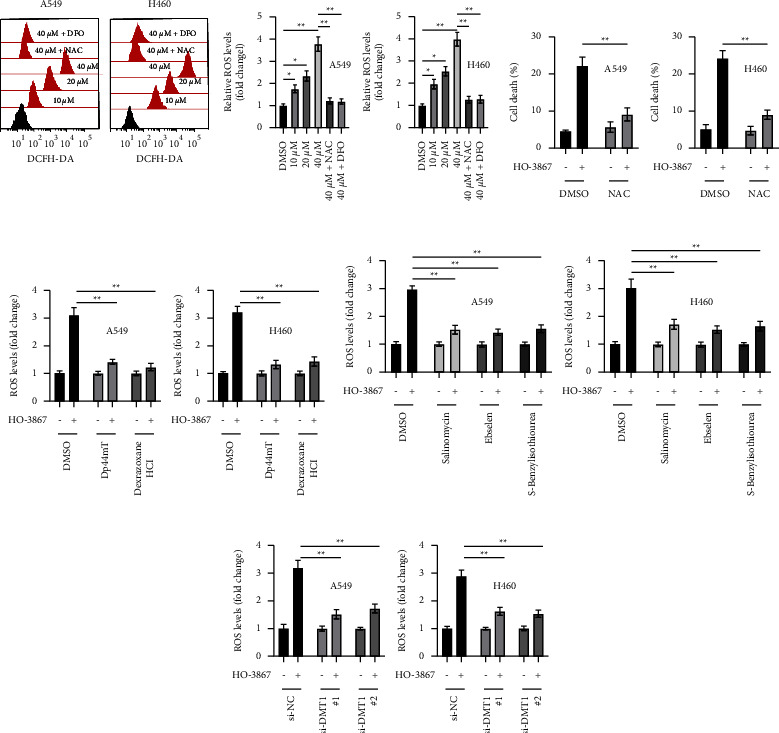
HO-3867 induced upregulation of ROS in an iron-dependent manner in NSCLC cells. (a) A549 and H460 cells were treated with indicated doses of HO-3867 alone or in combination with DFO (20 *μ*M) or NAC (10 *μ*M) for 24 h, and ROS levels were assayed. (b) A549 and H460 cells were incubated with or without HO-3867 (40 *μ*M) alone or in combination with NAC (10 *μ*M) for 24 h, and cellular death was assayed. (c) A549 and H460 cells were incubated with or without HO-3867 (40 *μ*M) alone or in combination with different iron chelators (Dp44mT: 20 *μ*M and dexrazoxane HCl: 15 *μ*M) for 24 h, and ROS levels were assayed. (d) A549 and H460 cells were incubated with or without HO-3867 (40 *μ*M) alone or in combination with different DMT1 inhibitors (salinomycin: 10 *μ*M, enselen: 20 *μ*M, and S-benzylisothiourea: 20 *μ*M) for 24 h, and ROS levels were assayed. (e) A549 and H460 cells were transfected as indicated for 24 h cells were treated with or without HO-3867 (40 *μ*M) for another 24 h, and ROS levels were assayed. Data are presented as mean ± SD. ^*∗*^*P* < 0.05; ^*∗∗*^*P* < 0.01.

The generation of ROS is another critical event in the process of ferroptosis [15]. In particular, we examined the difference in ROS levels in NSCLC cells both before and after they were exposed to HO-3867. As illustrated in Figure 6(a), a dosage-dependent elevation in ROS levels was observed in NSCLC cells when following the HO-3867 treatment. ROS scavenger (NAC) was capable of abrogating HO-3867's ability to produce an increase in ROS production in NSCLC cells (Figure 6(a)). NAC also remarkably suppressed HO-3867-mediated death of NSCLC cells (Figure 6(b)). Noteworthy, it was also observed that DFO attenuated HO-3867-triggered ROS upregulation in NSCLC cells (Figure 6(a)). To evaluate if HO-3867-induced upregulation of ROS is iron-dependent, more iron chelators were examined. The iron chelators Dp44mT and dexrazoxane HCl also reduced HO-3867-induced upregulation of ROS in NSCLC cells (Figure 6(c)). After that, we assessed the involvement of DMT1 in the HO-3867-triggered increase in ROS production in NSCLC cells. As can be seen in Figure 6(d), the levels of ROS produced by NSCLC cells decreased remarkably when DMT1 inhibitors were used. Similarly, the enhancement of ROS generation that was caused by HO-3867 in NSCLC cells was also decreased by DMT1 silencing (Figure 6(e)). Collectively, as evidenced by these data, it appears that HO-3867 causes an accumulation of ROS in NSCLC cells in a way that is reliant on iron.

### 3.7. HO-3867 Treatment Reduced GPX4 Expression in NSCLC Cells

**Figure 7:**
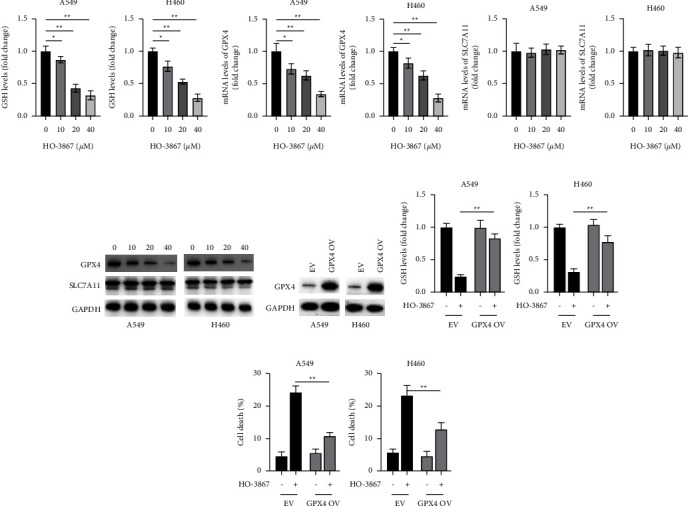
HO-3867 inhibited the expression of GPX4 in NSCLC cells. (a) A549 and H460 cells were treated with indicated doses of HO-3867 for 24 h, and GSH levels were assayed. (b) A549 and H460 cells were treated with the indicated doses of HO-3867 for 24 h, and mRNA levels of GPX4 were assayed by RT-PCR. (c) mRNA levels of SLC7A11 were assayed. (d) A549 and H460 cells were incubated with the indicated doses of HO-3867 for 24 h, and the indicated proteins were assayed by Western blotting. (e) A549 and H460 cells were transfected as indicated for 24 h, and protein levels of GPX4 were assayed. (f) A549 and H460 cells were transfected as indicated for 24 h, cells were treated with or without HO-3867 (40 *μ*M) for another 24 h, and GSH levels were assayed. (g) A549 and H460 cells were transfected as indicated for 24 h, then cells were treated with or without HO-3867 (40 *μ*M) for another 24 h, and cellular death was assayed. Data are presented as mean ± SD. ^*∗*^*P* < 0.01 and ^*∗∗*^*P* < 0.01.

The depletion of GSH is another essential hallmark of ferroptosis [15]. Thus, we examined the GSH level in NSCLC cells that had been pretreated with HO-3867. As can be seen in Figure 7(a), HO-3867 caused a dosage-dependent decrease in the level of GSH that was generated by NSCLC cells. SLC7A11 is a multipass transmembrane protein that regulates the transportation of cysteine, a rate-limiting precursor for the production of GSH [16]. Glutathione peroxidase 4 (GPX4) belongs to the GPX family of enzymes. It utilizes reduced GSH as a cofactor in its detoxification of lipid peroxide, thereby, inhibiting ferroptosis [17]. Thus, we evaluated how HO-3867 affected the concentrations of GPX4 and SLC7A11 in NSCLC cells. As per the outcomes of RT-PCR, HO-3867 contributed to a dosage-dependent decrease in the level of GPX4 mRNA found in NSCLC cells (Figure 7(b)). Even thorough HO-3867 did not have an impact on the levels of SLC7A11 mRNA in NSCLC cells (Figure 7(c)). Similar results were obtained using Western blotting, which showed that HO-3867 dosage-dependently decreased the protein levels of GPX4 in NSCLC cells, although it had no impact on the protein levels of SLC7A11 (Figure 7(d)). Overexpressing GPX4 in NSCLC cells allowed us to investigate whether or not it plays a role in the ferroptosis that is triggered by HO-3867 (Figure 7(e)). After being exposed to HO-3867, our findings showed that GPX4 overexpression increased GSH levels in NSCLC cells (Figure 7(f)). Furthermore, overexpression of GPX4 also protected NSCLC cells from death triggered by HO-3867. Thus, in NSCLC, the induction of ferroptosis by HO-3867 requires the downregulation of GPX4.

## 4. Discussion

Cell death is remarkably involved in many different physiological processes, including the progression of cancers and their subsequent treatments [5]. In this research, NSCLC cells were exposed to an analog of curcumin called HO-3867, which was then tested for its antitumor roles. We discovered that the use of HO-3867 decreased the viability of NSCLC cells with wild-type p53. Additionally, the HO-3867 treatment causes NSCLC cells to undergo both ferroptosis and apoptosis.

Extensive research has focused on apoptosis, a kind of cell death marked by caspase activation [18]. We established that HO-3867 induces apoptosis in NSCLC cells. At first, NSCLC cell death caused by HO-3867 was blocked by the pan-caspase inhibitor z.VAD.FMK. Second, caspase-3 activities assays showed that HO-3867 promoted the activities of caspase-3. Third, Western blotting revealed that HO-3867 induced caspase-3 cleavage. These results are consistent with past research demonstrating that HO-3867 triggers apoptosis in several cancer cells, such as those of the pancreas, ovaries, oral cavity, and osteosarcoma cells [19–22]. Apoptosis may occur through two different pathways, the intrinsic and extrinsic/mitochondrial pathways. The Bcl-2 family of proteins performs a fundamental function in modulating the intrinsic pathway [23]. We showed that the antiapoptotic Bcl-2 members Mcl-1 and Bcl-2 were downregulated in NSCLC cells after treatment with HO-3867. These data demonstrate that HO-3867 triggers apoptosis via the intrinsic mechanism, which is similar to earlier research that revealed that HO-3867 reduced Bcl-2 and Mcl-1 in ovarian cancer cells [24, 25]. Interestingly, another study found that HO-3867 upregulated Bcl-2 in cardiomyocytes [26]. This variation might be attributed to different cell types. Thus, understanding how HO-3867 regulates Bcl-2 proteins requires additional research.

Emerging research suggests that ferroptosis, a newly discovered mode of cell death, performs an instrumental function in modulating the advancement of many cancers, NSCLC included [27]. Ferroptosis is highly prevalent in lung cancer tissues because of increased levels of ROS and lipid peroxide [28]. Hence, to eradicate NSCLC cells, inhibiting ferroptosis could be an effective method. To date, extensive research has demonstrated that curcumin causes ferroptosis in various cancerous cells such as NSCLC and breast cancer cells [29, 30]. Here, we revealed, for the first time, that HO-3867 could also induce ferroptosis in NSCLC cells. Investigation of the mechanisms underlying HO-3867-induced ferroptosis revealed that HO-3867 activates the p53/DMT1 axis. Interestingly, HO-3867 has been illustrated to trigger wild-typep53-like effects by covalently binding to mutant p53 [31]. This suggests that HO-3867 may exert its effect, at least partially, via the regulation of p53. This may also explain why HO-3867 shows little cytotoxicity against p53-deficient cells. We found that HO-3867 caused the upregulation of p53 in NSCLC cells; however, another recent study showed that HO-3867 suppressed p53 levels in ovarian cancer cells [32]. This discrepancy reveals the complex effects of HO-3867 on the regulation of p53 and must be clarified.

Even though systemic iron dysregulation is prevalent in cancer patients, the link between iron and lung cancer risk remains controversial. For instance, several research reports have found that high dietary iron intake remarkably elevates the chance of developing lung cancer [33–35]. High iron intake has also been linked to a reduced chance of developing lung cancer [36]. Moreover, iron transportation-related proteins such as TFR, FTL, and FTH were found to be upregulated in lung cancer [37]. We observed that HO-3867 did not change the TFR, FTL, or FTH expression but did enhance the expression of DMT1. DMT1 works as a proton-coupled pump and uses the cell membrane potential for the active transport of iron [38]. Most cells ubiquitously express DMT1, with special prominence in the apical membrane of duodenal enterocytes [39]. Our findings suggest that DMT1 expression is subject to p53 regulation as the upregulation of DMT1 was abrogated in p53 deficiency cells. These findings were consistent with existing literature showing that p53 elevated DMT1 levels in kidney cell lines [40]. In another study, researchers observed that DMT1 expression in hepatocellular carcinoma cells was unaffected by p53 [41]. Hence, more investigations are necessary to further elucidate the link between p53 and DMT1. Moreover, we also discovered that DMT1 is essential for the buildup of ROS mediated by HO-3867. This is consistent with past research showing that inhibiting DMT1 in pancreatic *β* cells decreases ROS production and cell death [42]. Our findings shed new light on DMT1's function in NSCLC therapy.

Another vital finding of our study is that HO-3867 treatment reduced GPX4 levels in NSCLC cells. These data are in line with previous research that found curcumin-suppressed GPX4 levels in NSCLC and breast cancer cells [29, 30]. Remarkably, another research report found that ALZ003, another curcumin analog, also reduced GPX4 expression in glioblastoma cells [43]. Although we found that HO-3867 did not affect the expression of SLC7A11, curcumin is known to modulate the levels of SLC7A11 in lung cancer and lung epithelial cells [29, 44]. More research studies are warranted to clarify this disparity.

Nonetheless, there are a few drawbacks to this research. First, we did not investigate the anti-NSCLC effects of HO-3867 *in vivo* owing to constrained time and funding. Hence, the antitumor properties of HO-3867 should be investigated in xenograft models. Second, we found that Mcl-1 and Bcl-2 were both downregulated in response to HO-3867, but the mechanisms behind this phenomenon remain unclear. Also, we did not investigate the mechanisms underlying HO-3867-induced suppression of GPX4. HO-3867 is a STAT3 inhibitor [19]. Many studies suggest that STAT3 participates in the upregulation of GPX4 in various cells [45, 46]. Therefore, we hypothesized that HO-3867 downregulated GPX4 via inhibition of STAT3 and did not experimentally confirm it.

## 5. Conclusions

Our findings demonstrate that HO-3867, a curcumin analog, triggers both ferroptosis and apoptosis in p53 wild-type NSCLC cells. Mechanically, HO-3867 induces apoptosis by downregulating Bcl-2 and Mcl-1 levels and upregulating Bax. Also, HO-3867 activates the p53/DMT1 axis to increase iron accumulation, which further causes the accumulation of ROS in NSCLC cells. Moreover, HO-3867 also represses GPX4 in NSCLC cells. Collectively, these results imply that HO-3867 has the potential as a treatment target for NSCLC.

## Data Availability

The data used to support the findings of this study are available from the corresponding author upon request.

## References

[1] Siegel R. L., Miller K. D., Fuchs H. E., Jemal A. (2022). Cancer statistics, 2022. *CA: A Cancer Journal for Clinicians*.

[2] Gridelli C., Rossi A., Carbone D. P. (2015). Non-small-cell lung cancer. *Nature Reviews Disease Primers*.

[3] Herbst R. S., Morgensztern D., Boshoff C. (2018). The biology and management of non-small cell lung cancer. *Nature*.

[4] Hengartner M. O. (2000). The biochemistry of apoptosis. *Nature*.

[5] Dhokia V., Moss J. A. Y., Macip S., Fox J. L. (2022). At the crossroads of life and death: the proteins that influence cell fate decisions. *Cancers*.

[6] Tang D., Kang R., Berghe T. V., Vandenabeele P., Kroemer G. (2019). The molecular machinery of regulated cell death. *Cell Research*.

[7] Dixon S. J., Lemberg K. M., Lamprecht M. R. (2012). Ferroptosis: an iron-dependent form of nonapoptotic cell death. *Cell*.

[8] Wei X., Yi X., Zhu X. H., Jiang D. S. (2020). Posttranslational modifications in ferroptosis. *Oxidative Medicine and Cellular Longevity*.

[9] Qiu Y., Cao Y., Cao W., Jia Y., Lu N. (2020). The application of ferroptosis in diseases. *Pharmacological Research*.

[10] Wan Mohd Tajuddin W. N. B., Lajis N. H., Naidu R., Abas F., Othman I., Naidu R. (2019). Mechanistic understanding of curcumin’s therapeutic effects in lung cancer. *Nutrients*.

[11] Mohamadian M., Ahmadi S. S., Bahrami A., Ferns G. A. (2022). Review on the therapeutic potential of curcumin and its derivatives on glioma biology. *Neurochemical Research*.

[12] Selvendiran K., Kuppusamy M. L., Bratasz A. (2009). Inhibition of vascular smooth-muscle cell proliferation and arterial restenosis by HO-3867, a novel synthetic curcuminoid, through up-regulation of PTEN expression. *Journal of Pharmacology and Experimental Therapeutics*.

[13] Ravi Y., Sai-Sudhakar C. B., Kuppusamy P. (2021). PTEN as a therapeutic target in pulmonary hypertension secondary to left-heart failure: effect of HO-3867 and supplemental oxygenation. *Cell Biochemistry and Biophysics*.

[14] Yu R., Yu B. X., Chen J. F. (2016). Anti-tumor effects of Atractylenolide I on bladder cancer cells. *Journal of Experimental & Clinical Cancer Research*.

[15] Xie Y., Hou W., Song X. (2016). Ferroptosis: process and function. *Cell Death & Differentiation*.

[16] Koppula P., Zhang Y., Zhuang L., Gan B. (2018). Amino acid transporter SLC7A11/xCT at the crossroads of regulating redox homeostasis and nutrient dependency of cancer. *Cancer Communications*.

[17] Yang W. S., SriRamaratnam R., Welsch M. E. (2014). Regulation of ferroptotic cancer cell death by GPX4. *Cell*.

[18] Yao S., Ye J., Yin M., Yu R. (2020). DMAMCL exerts antitumor effects on hepatocellular carcinoma both in vitro and in vivo. *Cancer Letters*.

[19] Rath K. S., Naidu S. K., Lata P. (2014). HO-3867, a safe STAT3 inhibitor, is selectively cytotoxic to ovarian cancer. *Cancer Research*.

[20] Hu Y., Zhao C., Zheng H. (2017). A novel STAT3 inhibitor HO-3867 induces cell apoptosis by reactive oxygen species-dependent endoplasmic reticulum stress in human pancreatic cancer cells. *Anti-Cancer Drugs*.

[21] Chen C. W., Hsieh M. J., Ju P. C. (2022). Curcumin analog HO-3867 triggers apoptotic pathways through activating JNK1/2 signalling in human oral squamous cell carcinoma cells. *Journal of Cellular and Molecular Medicine*.

[22] Lu P. W. A., Chou C. H., Yang J. S. (2022). HO-3867 induces apoptosis via the JNK signaling pathway in human osteosarcoma cells. *Pharmaceutics*.

[23] Green D. R. (2022). The mitochondrial pathway of apoptosis Part II: the BCL-2 protein family. *Cold Spring Harbor Perspectives in Biology*.

[24] Selvendiran K., Ahmed S., Dayton A. (2011). HO-3867, a curcumin analog, sensitizes cisplatin-resistant ovarian carcinoma, leading to therapeutic synergy through STAT3 inhibition. *Cancer Biology & Therapy*.

[25] Terlikowska K. M., Witkowska A. M., Zujko M. E., Dobrzycka B., Terlikowski S. J. (2014). Potential application of curcumin and its analogues in the treatment strategy of patients with primary epithelial ovarian cancer. *International Journal of Molecular Sciences*.

[26] Dayton A., Selvendiran K., Meduru S. (2011). Amelioration of doxorubicin-induced cardiotoxicity by an anticancer-antioxidant dual-function compound, HO-3867. *Journal of Pharmacology and Experimental Therapeutics*.

[27] Xia X., Fan X., Zhao M., Zhu P. (2019). The relationship between ferroptosis and tumors: a novel landscape for therapeutic approach. *Current Gene Therapy*.

[28] Zhang Y., Guo R., Li J., Zhu L. (2022). Research progress on the occurrence and therapeutic mechanism of ferroptosis in NSCLC. *Naunyn-Schmiedeberg’s Archives of Pharmacology*.

[29] Tang X., Ding H., Liang M. (2021). Curcumin induces ferroptosis in non-small-cell lung cancer via activating autophagy. *Thorac Cancer*.

[30] Cao X., Li Y., Wang Y. (2022). Curcumin suppresses tumorigenesis by ferroptosis in breast cancer. *PLoS One*.

[31] Madan E., Parker T. M., Bauer M. R. (2018). The curcumin analog HO-3867 selectively kills cancer cells by converting mutant p53 protein to transcriptionally active wildtype p53. *Journal of Biological Chemistry*.

[32] Devor E. J., Schickling B. M., Lapierre J. R., Bender D. P., Gonzalez-Bosquet J., Leslie K. K. (2021). The synthetic curcumin analog HO-3867 rescues suppression of PLAC1 expression in ovarian cancer cells. *Pharmaceuticals*.

[33] Knekt P., Reunanen A., Takkunen H., Aromaa A., Heliovaara M., Hakuunen T. (1994). Body iron stores and risk of cancer. *International Journal of Cancer*.

[34] Yang W. S., Wong M. Y., Vogtmann E. (2012). Meat consumption and risk of lung cancer: evidence from observational studies. *Annals of Oncology*.

[35] Xue X. J., Gao Q., Qiao J. H., Zhang J., Xu C. P., Liu J. (2014). Red and processed meat consumption and the risk of lung cancer: a dose-response meta-analysis of 33 published studies. *International Journal of Clinical and Experimental Medicine*.

[36] Muka T., Kraja B., Ruiter R. (2017). Dietary mineral intake and lung cancer risk: the Rotterdam Study. *European Journal of Nutrition*.

[37] Zhang C., Zhang F. (2015). Iron homeostasis and tumorigenesis: molecular mechanisms and therapeutic opportunities. *Protein Cell*.

[38] Vogt A. C. S., Arsiwala T., Mohsen M., Vogel M., Manolova V., Bachmann M. F. (2021). On iron metabolism and its regulation. *International Journal of Molecular Sciences*.

[39] Gunshin H., Mackenzie B., Berger U. V. (1997). Cloning and characterization of a mammalian proton-coupled metal-ion transporter. *Nature*.

[40] Tong W. H., Sourbier C., Kovtunovych G. (2011). The glycolytic shift in fumarate-hydratase-deficient kidney cancer lowers AMPK levels, increases anabolic propensities and lowers cellular iron levels. *Cancer Cell*.

[41] Zhao N., Zhang A. S., Wortham A. M., Jue S., Knutson M. D., Enns C. A. (2017). The tumor suppressor, P53, decreases the metal transporter, ZIP14. *Nutrients*.

[42] Hansen J. B., Tonnesen M. F., Madsen A. N. (2012). Divalent metal transporter 1 regulates iron-mediated ROS and pancreatic beta cell fate in response to cytokines. *Cell Metabolism*.

[43] Chen T. C., Chuang J. Y., Ko C. Y. (2020). AR ubiquitination induced by the curcumin analog suppresses growth of temozolomide-resistant glioblastoma through disrupting GPX4-Mediated redox homeostasis. *Redox Biology*.

[44] Tang X., Li Z., Yu Z. (2021). Effect of curcumin on lung epithelial injury and ferroptosis induced by cigarette smoke. *Human & Experimental Toxicology*.

[45] Liu Q., Wang K. (2019). The induction of ferroptosis by impairing STAT3/Nrf2/GPx4 signaling enhances the sensitivity of osteosarcoma cells to cisplatin. *Cell Biology International*.

[46] Zhang W., Gong M., Zhang W. (2022). Thiostrepton induces ferroptosis in pancreatic cancer cells through STAT3/GPX4 signalling. *Cell Death & Disease*.

